# Transcriptome analysis reveals physiological responses in liver tissues of *Epinephelus cyanopodus* under acute hypoxic stress

**DOI:** 10.3389/fphys.2025.1697398

**Published:** 2025-12-09

**Authors:** Qiaoyi Chen, Yukun Huang, Zhiya Yu, Wenjie He, Xueqin Hu, Jinhui Wu, Tianguang Cai, Yuhua Cui, Along Gao, Hu Shu

**Affiliations:** 1 South China Biodiversity Research Center, College of Life Sciences, Guangzhou University, Guangzhou, Guangdong, China; 2 Agro-Tech Extension Center of Guangdong Province, Guangzhou, Guangdong, China; 3 Shenzhen Haijuyuan Aquacture Technology Co., Ltd., Shenzhen, Guangdong, China

**Keywords:** *Epinephelus cyanopodus*, hypoxic stress, RNA-Seq, metabolism, immune response

## Abstract

Dissolved oxygen (DO) in aquatic ecosystems plays a pivotal role in fish farming, serving as a critical determinant for the sustainable development of aquaculture practices. When fish suffer hypoxic stress, they undergo a cascade of physiological adaptations. In this study, healthy *E. cyanopodus* were subjected to experimental treatments under normoxic (6.0 ± 0.05 mg/L) and hypoxic (1.6 ± 0.05 mg/L) conditions for 1 (H1), 3 (H3), 6 (H6), and 9 (H9) h to evaluate physiological responses. Liver RNA-seq analysis identified 6152 differentially expressed genes (DEGs) between the control group (H0) and the four hypoxia-treated groups (H1, H3, H6, H9). RNA-seq results indicated that hypoxia for 3–6 h was the key duration when significant physiological changes occurred in *E. cyanopodus*. KEGG enrichment analysis revealed significant involvement of these DEGs in key hypoxia-responsive pathways, including HIF-1 signaling, Glutathione metabolism, p53 signaling, PPAR signaling, and PI3K-Akt signaling pathways. These DEGs primarily played function in biological processes, including glycolysis/gluconeogenesis (*aldob*, *hk*, *ldh-a*, *pparα*, *eno1*, *gpt*), pyruvate metabolism (*aldocb*, *ldh-a*, *fabp1*), immune response (*pnp*, *cxcl5*, *tnf-α*, *il1-β*, *il12-β*), and apoptosis regulation (*bax*, *bcl2*, *casp3*). Their coordinated expression played a crucial role in mediating hypoxic adaptation of the liver and brain in *E. cyanopodus*. Three immune-related enzymes (AKP, ALT, AST), and two metabolic-related enzymes (GLU, LDH) were significantly expressed at 3 and 6 h. These results exactly proved that 3–6 h of hypoxic stress was the key period when *E. cyanopodus* experienced significant physiological changes. This study elucidated key physiological response changes underlying hypoxic stress in *E. cyanopodus*, which provided both theoretical framework for understanding hypoxic adaptation and practical insights for developing hypoxia-resistant breeding strategies.

## 1 Introduction

In aquaculture, the aquatic environment constitutes a complex system influenced by numerous factors (Wu et al., 2024). While many variables influence aquatic systems, dissolved oxygen (DO) is especially critical. The concentration of DO in aquatic ecosystems is subject to dynamic variations, which are influenced by natural factors such as seasonal shifts, photosynthesis, wind, salinity, temperature changes, and diurnal cycles (Chandel et al., 1997; Ma et al., 2013). Moreover, in intensive aquaculture systems, high-density farming practices result in the accumulation of unconsumed feed and fertilizers (Mader et al., 2017; Saetan et al., 2020). Besides, the natural environment continues to deteriorate, particularly due to agricultural run-off caused by excessive use of fertilizer and pesticide. These consequences promote excessive phytoplankton proliferation, triggering harmful algal blooms such as red tides. It accelerates water eutrophication which further deplete DO and exacerbate hypoxic conditions (Lefevre et al., 2014; Li et al., 2018). This phenomenon poses a significant threat to the health and sustainability of aquaculture (Mu et al., 2020; Sagasti et al., 2001). Consequently, fish have evolved complex physiological mechanisms to maintain oxygen homeostasis under hypoxic stress (Gong et al., 2024). These adaptive responses including metabolic modulation, immune system adjustments, and a suite of regulatory adaptations, such as a metabolic shift from aerobic glycolysis to anaerobic glycolysis/gluconeogenesis and pyruvate metabolism, elevating respiratory rate, enhancing angiogenesis, increasing erythrocyte production, alongside an activated immune response to enhance hypoxic tolerance (Zhao et al., 2022). Existing research has demonstrated that the molecular mechanisms underlying hypoxic adaptation in fish primarily involve energy utilization, oxygen transport efficiency, glucose metabolism regulation, and hypoxic resistance pathways (Aragonés et al., 2009; Chen et al., 2017; Gong et al., 2020; Hammarlund et al., 2020). These findings collectively provide crucial insights into the regulatory frameworks governing hypoxic stress responses in aquatic species.

*Epinephelus cyanopodus*, also known as the Speckled blue grouper, is a marine species of the Western Pacific, is classified within the Osteichthyes class, order Perciformes, family Serranidae, and genus *Epinephelus*. It is highly valued as a commercial species, which represents an important economic resource in Chinese fisheries sector (Cao et al., 2022). It is also prized for its high-quality muscle tissue, which contains abundant unsaturated fatty acids, particularly including eicosapentaenoic acid (EPA) and docosahexaenoic acid (DHA), while maintaining low fat (Cai et al., 2025). In recent years, *E. cyanopodus* farming has experienced rapid expansion. However, research on premium species such as *E. cyanopodus* remains relatively underdeveloped, with numerous challenges persisting. A key issue in intensive farming systems is the high-density cultivation environment. Although this approach offers greater economic returns for *E. cyanopodus* production, it also generates more pronounced adverse effects, particularly the reduction and fluctuation of DO levels in rearing waters (Sun et al., 2011). Acute severe hypoxia (DO < 1.0 mg/L) poses significant risks to most fish, including surfacing behavior (floating head), cessation of feeding, severely damaged the immune system, and even caused mortality (Domenici et al., 2007; Richards, 2011). These challenges pose significant constraints on the sustainable development of the *E. cyanopodus* aquaculture farming. To ensure the long-term viability of this industry, it is urgent to investigate the physiological adaptations to hypoxia and elucidate the underlying molecular regulatory mechanisms.

Transcriptome sequencing (RNA-seq) is a comprehensive study of gene expression at the RNA level and provides information on differentially expressed genes (DEGs) and gene functions. Nowadays, RNA-seq is now widely used in aquaculture as a standard method for analyzing DEGs (Xu et al., 2015; Zhang et al., 2022). Moreover, this approach is able to distinguish diverse gene expression patterns, facilitate the development of novel selective markers, and contribute to the discovery of previously uncharacterized genes (Herkenhoff et al., 2018; Jin et al., 2013). RNA-seq of liver has served as a valuable tool for elucidating hypoxic tolerance mechanisms in Osteichthyes, such as *Epinephelus coioides* (Wu et al., 2023), Pearl gentian grouper (*Epinephelus lanceolatus* ♂ × *Epinephelus fuscoguttatus* ♀) (Liang et al., 2024; Wang et al., 2024), *Micropterus salmoides* (He et al., 2022), *Ctenopharyngodon idella* (Zhang et al., 2025), *Oreochromis niloticus* (Ma et al., 2021). However, the molecular mechanisms underlying hypoxic adaptation in *E. cyanopodus* remain poorly explored, particularly through transcriptomic approaches.

This study aimed to elucidate the molecular response of *E. cyanopodus* to acute hypoxic stress through RNA-seq analysis. We conducted RNA-seq on liver tissues across multiple hypoxic exposure timepoints to identify critical hypoxia-responsive genes and pathways. Quantitative real-time PCR (qRT-PCR) was employed to validate hypoxia-responsive candidate genes and the expression patterns of key hypoxic genes in liver and brain. This study elucidated how energy metabolism, immune response, and cellular apoptosis influenced hypoxic tolerance by detecting gene expressions and enzyme activities in *E. cyanopodus*, providing not only a theoretical framework for breeding hypoxia-resistant varieties, but also insights applicable to understand hypoxic regulation mechanisms in other marine fish species under intensive aquaculture conditions.

## 2 Materials and methods

### 2.1 Ethical statement

The handling procedures and experimental protocols of all animals in this experiment were in accordance with the Experimental Animal Ethics Committee of Guangzhou University of China [(2025) 009].

### 2.2 Experimental fish and acute hypoxic exposure

The experiment was accomplished in Guangdong Marine Fishery Experiment Center. Healthy *E. cyanopodus* (average standard weight: 5.6 ± 0.3 g; average standard length: 6.1 ± 0.2 cm) were acclimated in cylindrical tanks (diameter: 1 m, height: 1.2 m) with constant ventilation for 2 weeks before prescription. The salinity of water was 20–30 ppt, the temperature was 28 °C ± 1 °C and the DO was 6.0 ± 0.05 mg/L. During the acclimatization period, fish were fed with commercial pellet feeds twice a day, the circulating seawater was used to maintain normal physiological activities of *E. cyanopodus*.

In order to determine the semi-lethal concentration of DO, 150 *E. cyanopodus* were collected in 5 preliminary experiment buckets with 30 fish in each bucket. We reduced the volume of water to one-third of the buckets to make oxygen consumption faster. In addition, the supply of oxygen was cut off by stopping aeration, reducing the supply of circulating water and covering the buckets with plastic films (Lai Xing-xing et al., 2022). The DO of water was respectively reduced to 0.9, 1.2, 1.5, 1.8, and 2.1 mg/L within 1 h, and the change of DO was monitored by a dissolved oxygen detector (Leici, Shanghai, China). Under the hypoxic stress, *E. cyanopodus* moved slowly, their bodies darkened, showed visible spots, floated unsteadily and lost balance. The numbers of dead *E. cyanopodus* were recorded at 1, 3, 6, and 9 h under each DO concentration treatment (Liang et al., 2022). Within 9 h, the cumulative mortality of *E. cyanopodus* was calculated under each treatment. Finally, the semi-lethal concentration of DO in 9 h was found to be 1.59 mg/L by linear interpolation (Song et al., 2022). In the subsequent hypoxic stress experiment, a concentration of 1.6 ± 0.05 mg/L of DO was used as the semi-lethal concentration for *E. cyanopodus*.

In total, 120 healthy individuals were divided into 3 buckets as 3 parallel buckets with 40 fish in each bucket, and all the experimental fish had been fasted for 24 h before the experiment. At the beginning of the experiment, we randomly took 3 fish from each bucket under normoxic condition, which was named as H0. Then the DO of the water in each bucket was reduced from 6.0 ± 0.05 mg/L to 1.6 ± 0.05 mg/L within 1 h. Throughout the hypoxic stress experiment, the concentration of DO was maintained at 1. 6 ± 0.05 mg/L by adjusting the input of oxygen and water. Under this condition of maintaining the DO level, 3 fish were randomly taken from each bucket at 1, 3, 6, and 9 h, respectively, and recorded as hypoxic stress groups H1, H3, H6 and H9 (Liang et al., 2024). All the experimental fish were anesthetized and then dissected. 45 liver and 45 brain tissues were obtained and collected into a centrifuge tube equipped with RNA Keeper Tissue Stabilizer (Vazyme, Nanjing, China). The liver tissues which were used for enzyme activity detection were cleaned by PBS and placed directly into an empty centrifuge tube and immediately stored in liquid nitrogen.

### 2.3 RNA extraction, cDNA library construction and sequencing

Total RNA was isolated from samples using Trizol reagent (thermofisher, 15596018) according to the manufacturer’s protocol. Specifying the thresholds or criteria used for assessing RNA quantity and purity by using Bioanalyzer 2100 and RNA 6000 Nano LabChip Kit (Agilent, CA, United States). Only high-quality RNA samples (RIN number >7.0) were used for sequencing library construction. The cleaved RNA fragments were subsequently reverse-transcribed into cDNA using SuperScript™ II Reverse Transcriptase (Invitrogen, CA, United States). After UDG enzyme (NEB, cat.m0280, USA) treatment of U-labeled second-strand DNA, the ligated products were PCR-amplified under the following conditions: initial denaturation at 95 °C for 3 min; 8 cycles of 98 °C for 15 s, annealing at 60 °C for 15 s, and extension at 72 °C for 30 s; and a final 72 °C for 5 min. The final cDNA libraries had an average insert size of 300 ± 50 bp. Three biological replicate samples were set up in each group. At last, we performed the 2 × 150 bp paired-end sequencing (PE150) on an Illumina NovaseqTM 6000 following the vendor’s recommended protocol.

### 2.4 Sequence concatenation and functional annotation

Cutadapt (1.9) was used to filter reads containing adapter contamination, low-quality bases, or undetermined bases. Sequence quality was then assessed with FastQC (http://www.bioinformatics.babraham.ac.uk/projects/fastqc/, 0.10.1), including Q20, Q30, and GC-content metrics. All downstream analyses used high-quality clean data. *De novo* assembly of the transcriptome was performed by Trinity (2.15) without reference genomes. The longest transcript within each cluster was selected as the representative ‘gene’ sequence (designated as Unigene). Gene expression levels were quantified using Salmon (v1.9.0) through TPM calculation (Transcripts Per Kilobase of exon model per Million mapped reads).

### 2.5 Identification and enrichment analysis of differentially expressed genes

All assembled Unigenes were aligned against the Gene ontology (GO) (http://www.geneontology.org) and Kyoto Encyclopedia of Genes and Genomes (KEGG) (http://www.genome.jp/kegg/) databases using DIAMOND (2.0.15) with a threshold of E-value threshold of <0.00001. Gene differential expression analysis was performed by edgeR (3.40.2) software between two different groups and two different samples. Genes with a false discovery rate (FDR) <0.05 and fold change >2 or <0.5 were considered significantly differentially expressed. NormqPCR (1.44.0) was used to evaluate the housekeeping gene, which was subsequently used as a control for qRT-PCR.

### 2.6 Quantitative real-time PCR (qRT-PCR) validation

To validate RNA-seq results, 23 genes were selected for qRT-PCR analysis. Specific primers were designed using the mRNA sequences of *E. cyanopodus* via the NCBI Primer-BLAST tool (https://www.ncbi.nlm.nih.gov/). Primers for qRT-PCR are listed in Table 1, with *β-actin* and *ef1α* as the internal reference genes. qRT-PCR was performed on a LightCycler® 480 Instrument II (Roche, Switzerland) by using 384-well plate. The total reaction mixture system was 12.5 μL, containing 6.25 μL SYBR qPCR Master Mixture (Yeasen, Shanghai, China), 4.25 μL RNase-free ddH2O, both the forward and reverse primers were 0.5 μL, 1 μL cDNA (concentration 10 ng/μL). The qPCR reaction condition was as follows: preheating was set at 95 °C for 30 s, followed by 40 cycles of 95 °C for 10 s, 60 °C for 30 s. The melting curve was generated using the instrument default values of 95 °C for 15 s, 60 °C for 60 s, and 95 °C for 15 s to detect non-specific amplification and primer dimer (Lai Xingxing et al., 2022). Each experiment was repeated independently at least three times. In this experiment, the expression levels of the targeted genes were calculated by using the 2^−ΔΔCt^ method (Zhong et al., 2021).

**TABLE 1:** Specific primers of 23 genes for qRT-PCR.

Primer name	Forward primer (5′-3′)	Reverse primer (5′-3′)
*foxo1α*	ACAGCCGCTTTATCCGTGTC	GCACAAAAGCATTAGCTGGATT
*ddit4*	ATTGCGTCTTCTCAACAGGGT	GTTTCTCGGGGATGAGCAGT
*hif1α*	ACCCTCGTCTGTATCGCCTT	TGATCGGTCGGTTACCTCCA
*pnp*	ACTCCTTGATAAGCTTCGTGC	AACCCTGGGGTACACCTCTT
*ccnd1*	AGACGGCATGCAGAAGACAT	GCATTTAGGGGGAAGGAGCA
*bmp7*	AGACGGCATGCAGAAGACAT	GCATTTAGGGGGAAGGAGCA
*igfbp5*	ACGAGAAGCTGACTTCCGTG	GAACAGGTAGCACAGCGGAT
*gadd45β*	TTCTCCTTCCTTCCTCCCCAG	GACTCGCAGGATGGTAATGC
*foxo4*	TGCTCACCTCTGATTCCCCT	TGGTTGGGTTTGGACCTCAC
*pparα*	CTGGCAGAGAGGACGTTAGC	TTCCGTCAACTCTGTGACCG
*nfkbia*	TCACCTATGGTCGCACCAAC	CTCTTCCTCGCTGTCGTCTG
*cyp24a1*	GCTGCAGCACTTCAAACCAA	CAGTGCCTCGTGTTGTCTCA
*aldob*	AACCTGAACGCCATCAACCA	TTAGCCCTGGTGCAGAAAGC
*aldocb*	CACAGACGATGGCACTTCCT	CGCCGTCCTTCTTGTACTGT
*bcl2*	GACTGTACCAGCCGGACTTC	ATAATCCGGCCCCAGTTCAC
*bax*	GACGCAAGGATAGGAGAGGC	CTCCTTGTCGTCTTGGACCC
*casp3*	ACCAGTCAGTCGAGCAGTTG	CAGCGATCGCCTCGAAAAAG
*il1b*	GTGAGGTCGATGCAACAGGA	CGCAGTGAGTCAGGGACTTT
*eno1*	CCAGGATGACTGGGACGCAT	GGTTGACTTTAAGCAGCAGGC
*hk*	TCTTCACAAGACAGTCCGCC	CGGAATTCGGACAGCGTTTC
*ldha*	CAGTGTGACAGCCAACTCCA	AGTTGGGGCTGTACTTGACG
*ef1α*	GTAAGGAGGGCAATGCCAGT	CGTACCGGGCTTCAGGATAC
*β-actin*	TACGAGCTTCCTGACGGACA	GGCTGTGATCTCCTTCTGC

### 2.7 Enzyme activity detection

The liver tissues used for enzyme activity detection were mixed with a certain amount of PBS according to the kit instructions, then centrifuged at 8000 rpm for 10 min at 4 °C. Three techniques were used to detect each enzyme in each sample. The samples were pretreated according to the kit instructions. The 5 indexes including lactate dehydrogenase (LDH: A020-2-2), alkaline phosphatase (AKP: A059-2-2), alanine aminotransferase (ALT: C009-2-1), aspartate aminotransferase (AST: C010-2-1) and glucose (GLU: A154-1-1) were completed on the 96-well cell plate, and 5 indexes were detected by multifunctional enzyme labeling instrument (Infinite® 200PRO, Switzerland). Total protein (TP: A045-2-2) was detected by UV-visible spectrophotometer (Youke; Shanghai, China). All kits were obtained from Nanjing Jiancheng Bioengineering Institute (Nanjing, China).

### 2.8 Statistical analysis

Statistical analysis was performed using SPSS Statistics v27. One-way ANOVA followed by *post hoc* tests (Tukey’s) was used to assess differences among groups. The calculation method was selected by t-test, and the difference of *p* < 0.05 in homogeneous dataset was used for mapping. Significant differences are indicated by an asterisk (*) when plotting with GraphPad Prism v9.5 software.

## 3 Results

### 3.1 Semi-lethal concentration of dissolved oxygen in *E. cyanopodus*

**FIGURE 1:**
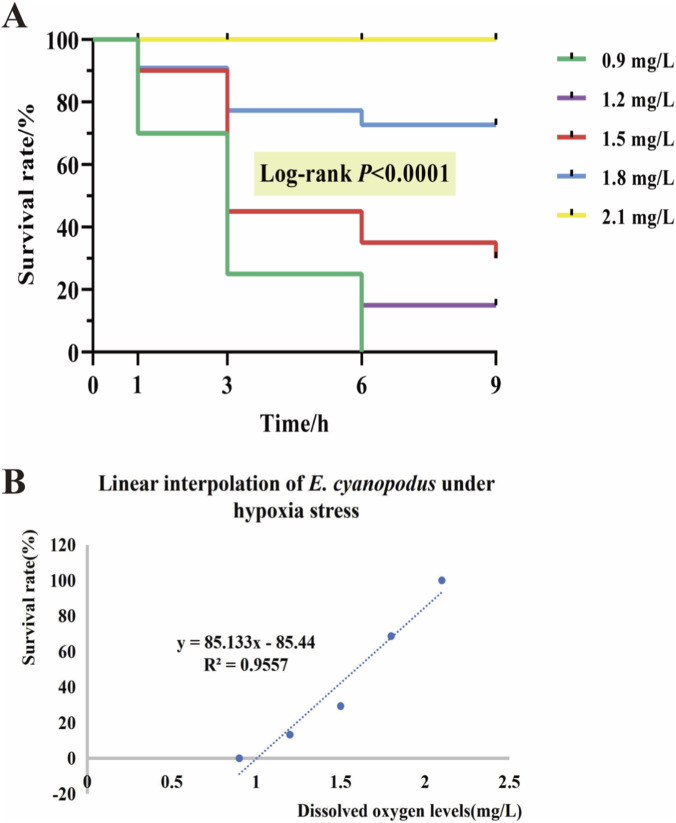
Survival curves of *E. cyanopodus* under different concentrations of hypoxic stress within 9 h **(A)**. Linear interpolation diagram of survival rate of *E. cyanopodus* under different concentrations of hypoxic stress within 9 h **(B)** (*n* = 30).

As shown in Table 2, with the continuous decreased in DO concentration (treatment temperature = 28 °C), the survival rate of *E. cyanopodus* also decreased. Survival curves of *E. cyanopodus* under different hypoxic concentrations over 9 h were generated according to the survival rate (Figure 1A). The DO levels and survival rate within 9 h were respectively taken as independent and dependent variables, and the regression equation was obtained by linear interpolation method: y = 85.133 x-85.44 (*R* ^2^ = 0.9557) (Figure 1B). Finally, the semi-lethal dissolved oxygen concentration of *E. cyanopodus* within 9 h was determined to be 1.59 mg/L, a concentration of 1.6 ± 0.05 mg/L of DO was conducted in the subsequent experiment.

**TABLE 2:** Effects of different concentrations of hypoxic stress on the survival rate of *E. cyanopodus* within 9 h.

Time (h)Survival rate (%)	DO (mg/L)/(T = 28 °C)				
	0.9	1.2	1.5	1.8	2.1
0	100	100	100	100	100
1	68.7	65.3	88.3	88.3	100
3	23.3	24.7	43.7	77.7	100
6	0	13.3	35.3	72.7	100
9	0	13.3	29.3	69.7	100

### 3.2 Quality of RNA-seq data

By transcriptome sequencing of 15 liver samples of *E. cyanopodus*, a total of 652,783,748 raw reads (97.93 G) were obtained from the 5 groups, The size range of cleaned reads in each library was 5.22 G to 8.32 G. The Q20 and Q30 values were respectively 97.26%–98.33% and 91.87%–95.03%. The GC content of all samples ranged from 49.99% to 51.70% (Table 3). These results indicated high reproducibility between samples within the group, and the obtained high quality transcriptome data could be used for further analysis.

**TABLE 3:** Summary of RNA-seq data of *E. cyanopodus*.

Sample	Raw_Reads	Raw_Bases	Valid_Reads	Valid_Bases	Valid%	Q20%	Q30%	GC%
H0-1	57,352,878	8.60G	54,972,328	8.06G	95.85	98.28	94.93	50.04
H0-2	40,435,326	6.07G	38,763,604	5.68G	95.87	97.46	92.40	50.50
H0-3	44,692,908	6.70G	43,111,700	6.32G	96.46	97.41	92.24	49.76
H1-1	38,904,204	5.84G	37,760,748	5.56G	97.06	98.10	94.14	50.04
H1-2	44,498,166	6.67G	42,890,974	6.28G	96.39	97.47	92.47	50.38
H1-3	38,578,202	5.79G	36,958,704	5.39G	95.80	97.98	93.97	49.74
H3-1	36,371,700	5.46G	35,387,862	5.22G	97.30	98.09	93.77	49.70
H3-2	58,619,446	8.79G	56,598,716	8.32G	96.55	98.33	95.03	49.49
H3-3	48,176,582	7.23G	46,406,522	6.80G	96.33	97.47	92.33	49.53
H6-1	45,703,374	6.86G	44,165,146	6.47G	96.63	97.31	91.93	49.75
H6-2	39,388,178	5.91G	37,781,230	5.53G	95.92	97.35	92.03	49.67
H6-3	37,161,016	5.57G	36,031,570	5.31G	96.96	98.01	93.54	49.83
H9-1	48,100,434	7.22G	45,831,460	6.68G	95.28	97.26	91.87	51.70
H9-2	36,532,026	5.48G	35,627,136	5.24G	97.52	97.58	92.90	49.75
H9-3	38,269,308	5.74G	37,264,958	5.47G	97.38	97.56	92.85	50.01

### 3.3 Summary of DEGs between groups

**FIGURE 2:**
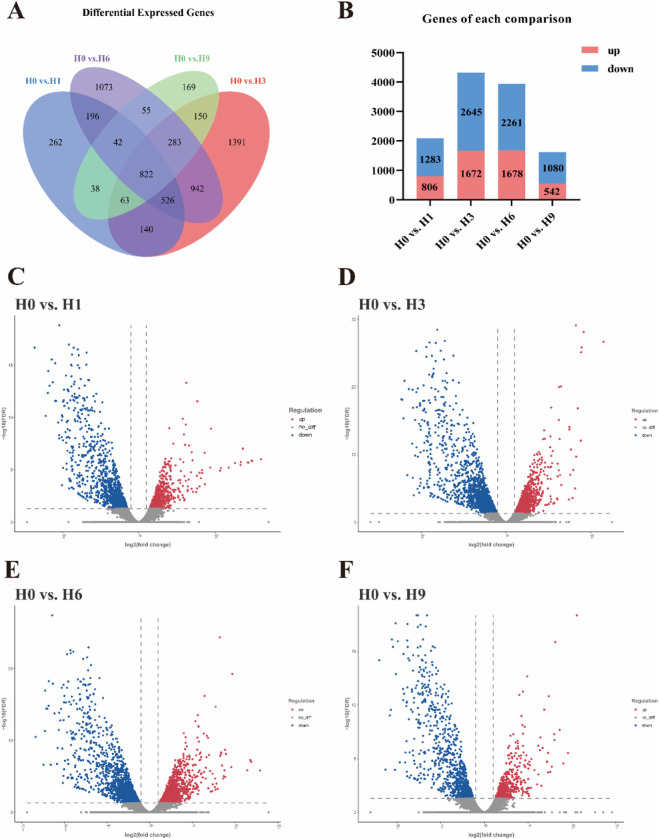
Venn diagram **(A)** of DEGs in the four comparisons, and numbers of upregulated and downregulated DEGs in each comparison **(B)**, volcano diagram of H0 vs. H1 **(C)**, volcano diagram of H0 vs. H3 **(D)**, volcano diagram of H0 vs. H6 **(E)**, and volcano diagram of H0 vs. H9 **(F)**.

In total, 6152 DEGs showed differential expression between the control group (H0) and the four hypoxic stress groups (H1, H3, H6, and H9). The Venn diagram showed a total of 822 DEGs between four comparisons, 914 DEGs between three comparisons and 1521 DEGs between two comparisons (Figure 2A). In H0 vs. H1, H0 vs. H3, H0 vs. H6, and H0 vs. H9 respectively contained 2089 DEGs (806 upregulated and 1283 downregulated) (Figures 2B,C), 4317 DEGs (1672 upregulated and 2645 downregulated) (Figures 2B,D), 3939 DEGs (1678 upregulated and 2261 downregulated) (Figures 2B,E) and 1622 DEGs (542 upregulated and 1080 downregulated) (Figures 2B,F). It was worth mentioning that the H0 vs. H3 comparison had the largest number of DEGs, in which the downregulated genes accounted for 61.27%; The H0 vs. H6 comparison had the second largest number of DEGs, with 57.40% of downregulated genes (Figure 2B). Therefore, it could be considered that acute hypoxia for 3 h and 6 h might be the two key time points of *E. cyanopodus*.

### 3.4 GO enrichment analysis of DEGs

**FIGURE 3:**
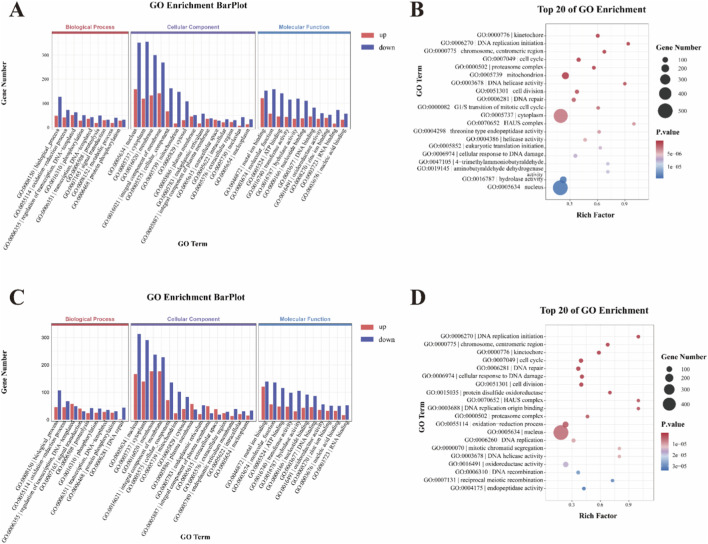
GO enrichment analysis of DEGs in H0 vs. H3 **(A)** and H0 vs. H6 **(C)**. The top 20 pathways in GO enrichment analysis of DEGs in H0 vs. H3 **(B)** and H0 vs. H6 **(D)**.

Among the three main GO functional enrichment analysis had revealed that, the majority of DEGs in both H0 vs. H3 and H0 vs. H6 were downregulated (Figures 3A,C). Most of the DEGs were clustered into the three main functional categories of biological process (BP), cellular component (CC) and molecular function (MF), with 35 subcategories, including 9 BP terms, 15 CC terms, and 11 MF terms, where the largest subcategories in the BP, CC and MF groups were “biological processes,” “nucleus” and “metal ion binding,” respectively.

In the GO enrichment analysis, there were 4317 DEGs in the H0 vs. H3 comparison. The most enriched terms in the BP functional category were biological processes (GO:0008150) and oxidation-reduction processes (GO:0055114). The most enriched terms in the CC functional category were nucleus (GO: 0005634), cytoplasm (GO: 0005737), and membrane (GO: 0016020). Metal ion binding (GO: 0046872), molecular function (GO: 0003674), and ATP binding (GO: 0005524) were the three main terms in the MF functional category (Figure 3A). The top 20 most-enriched GO terms were mainly enriched in the BP and CC function categories, such as Kinetochore (GO:0000776), DNA replication initiation (GO:0006270), cell cycle (GO:0007049), proteasome complex (GO:0000502), mitochondria (GO:0005739) (Figure 3B).

In the GO enrichment analysis, there were 3939 DEGs in the H0 vs. H6 comparison. The most enriched terms in the BP functional category were biological processes (GO: 0008150) and oxidation-reduction processes (GO:0055114). The most enriched three terms in the CC functional category were nucleus (GO: 0005634), cytoplasm (GO: 0005737), and membrane (GO: 0016020). Metal ion binding (GO: 0046872), molecular function (GO: 0003674), and ATP binding (GO: 0005524) were the three main terms in the MF functional category (Figure 3C). The top 20 most-enriched GO terms were mainly enriched in the BP and CC function categories. Examples including DNA replication initiation (GO:0006270), Kinetochore (GO:0000776), cell cycle (GO:0007049), DNA repair (GO:0006281), and cellular response to DNA damage (GO:0006974) (Figure 3D).

### 3.5 KEGG enrichment analysis of DEGs

**FIGURE 4:**
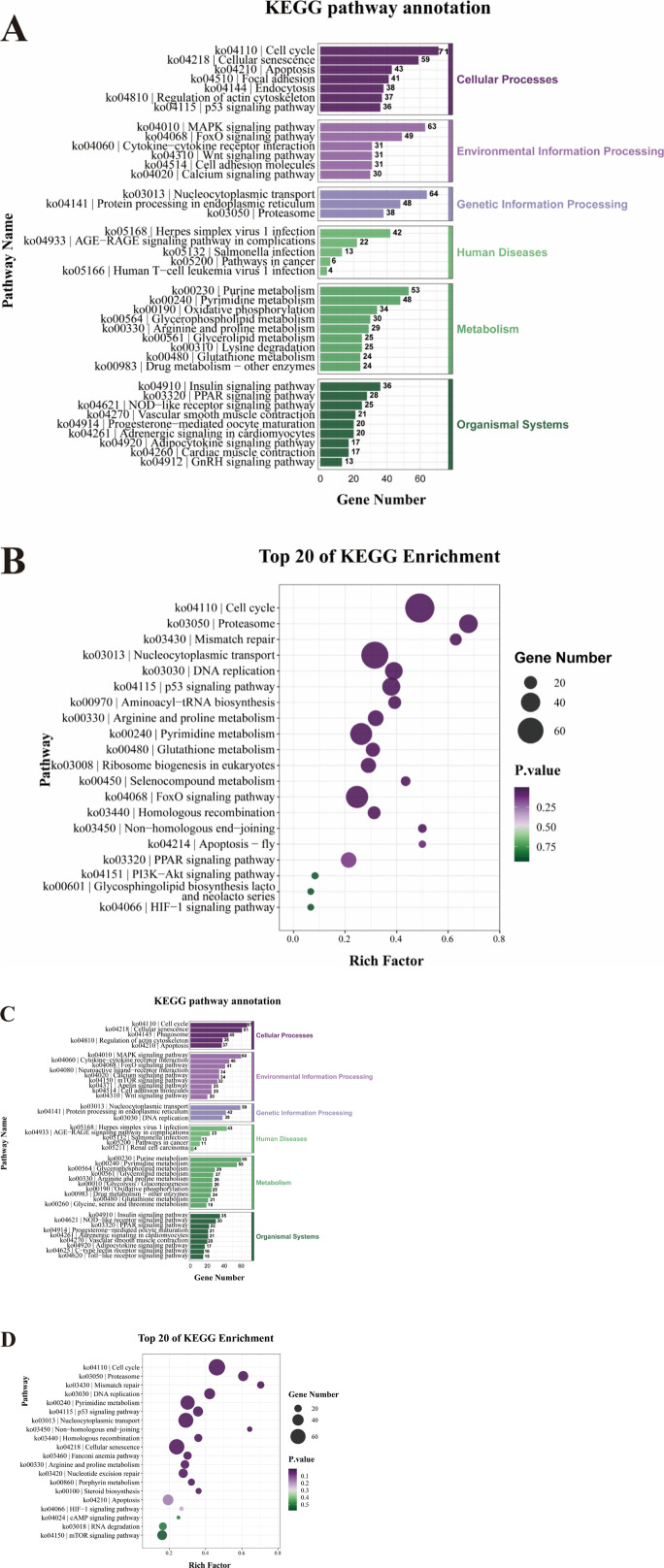
KEGG enrichment analysis of DEGs in H0 vs. H3 **(A)** and H0 vs. H6 **(C)**. The top 20 pathways in KEGG enrichment analysis of DEGs in H0 vs. H3 **(B)** and H0 vs. H6 **(D)**.

According to KEGG pathway enrichment analysis, 1286 DEGs identified in H0 vs. H3 were significantly enriched in 39 pathways distributed across six functional categories, including cellular processes (325 DEGs in 7 pathways), environmental information processing (235 DEGs in 6 pathways), genetic information processing (150 DEGs in 3 pathways), human diseases (87 DEGs in 5 pathways), metabolism (292 DEGs in 9 pathways), and organismal systems (197 DEGs in 9 pathways) (Figure 4A). Among the top 20 most-enriched KEGG terms, the pathways most associated with hypoxic stress were p53 signaling pathway (ko04115), HIF-1 signaling pathway (ko04066), Glutathione metabolism signaling pathway (ko00480), FoxO signaling pathway (ko04068), and PI3K-Akt signaling pathway (ko04151) (Figure 4B).

There were 1307 DEGs in H0 vs. H6 were significantly enriched in 41 pathways across six functional categories, including cellular processes (248 DEGs in 5 pathways), environmental information processing (317 DEGs in 9 pathways), genetic information processing (139 DEGs in 3 pathways), human diseases (94 DEGs in 5 pathways), metabolism (312 DEGs in 10 pathways), and organismal systems (197 DEGs in 9 pathways) (Figure 4C). Among the top 20 most-enriched KEGG terms, the pathways most associated with hypoxic stress including p53 signaling pathway (ko04115), HIF-1 signaling pathway (ko04066), cAMP signaling pathway (ko04024), mTOR signaling pathway (ko04150), and Apoptosis signaling pathway (ko04210) (Figure 4D).

### 3.6 Key DEGs involved in hypoxic response

**FIGURE 5:**
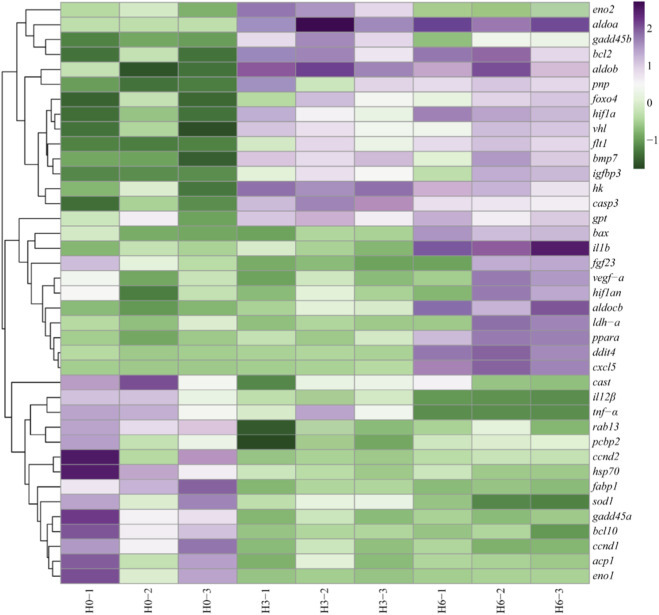
Heat map of hypoxic responsive DEGs under acute hypoxic stress at 0 h, 3 h, 6 h. Purple indicates gene upregulated expression, green indicates gene downregulated expression.

In order to better determine the changes of DEGs expression in *E. cyanopodus* under hypoxic conditions, the DEGs in the livers that were associated with hypoxia were identified. Among the enriched DEGs, 39 DEGs were associated with hypoxic reactions, and the expression levels of these DEGs in the H0, H3, and H6 groups were shown in the heat map (Figure 5). Most of these DEGs were enriched in the PI3K-Akt signaling pathway, p53 signaling pathway, mTOR signaling pathway, MAPK signaling pathway. These pathways were mainly involved in angiogenesis, metabolism, cell proliferation and growth, regulation of cell apoptosis.

Compared with H0 group, 14 DEGs in the H3 and H6 groups were significantly downregulated, such as calpastatin (*cast*), interleukin 12-beta (*il12β*), tumor necrosis factor-alpha (*tnf-α*), ras-related protein rab-13 (*rab13*), poly (RC) binding protein 2 (*pcbp2*), cyclin D2 (*ccnd2*), heat shock protein 70 (*hsp70*), fatty acid binding protein 1 (*fabp1*), superoxide dismutase 1 (*sod1*), growth arrest and DNA damage inducible alpha (*gadd45α*), B-cell lymphoma-10 (*bcl10*), cyclin D1 (*ccnd1*), acid phosphatase 1 (*acp1*), enolase 1 (*eno1*). In the H3 group, 15 DEGs were significantly upregulated, such as enolase 2 (*eno2*), aldolase, fructose-bisphosphate A (*aldoa*), growth arrest and DNA damage inducible beta (*gadd45β*), B-cell lymphoma-2 (*bcl2*), aldolase, fructose-bisphosphate B (*aldob*), purine nucleoside phosphorylase (*pnp*), forkhead box o4 (*foxo4*), hypoxia inducible factor 1 subunit alpha (*hif1α*), von hippel-lindau tumor suppressor (*vhl*), fms related receptor tyrosine kinase 1 (*flt1*), bone morphogenetic protein 7 (*bmp7*), insulin like growth factor binding protein 3 (*igfbp3*), hexokinase (*hk*), caspase 3 (*casp3*), glutamic-pyruvic transaminase 2 (*gpt*). In the H6 group, 24 DEGs were significantly upregulated, such as *aldoa*, *gadd45β*, *bcl2*, *aldob*, *pnp*, *foxo4*, *hif1α*, *vhl*, *flt1*, *bmp7*, *igfbp3*, *hk*, *casp3*, *gpt*, BCL-2 associated X (*bax*), interleukin 1 Beta (*il1b*), fibroblast growth factor 23 (*fgf23*), vascular endothelial growth factor A (*vegf-a*), hypoxia inducible factor 1 subunit alpha inhibitor (*hif1an*), aldolase C-fructose-bisphosphate-b (*aldocb*), lactate dehydrogenase A (*ldh-a*), peroxisome proliferator activated receptor alpha (*pparα*), DNA damage inducible transcript 4 (*ddit4*), C-X-C motif chemokine ligand 5 (*cxcl5*).

In H3 and H6 groups, metabolism related genes (*hk*, *ldh-a*, *pparα* and *foxo4*), anaerobic glycolytic related genes (*aldocb* and *ldh-a*), apoptosis related gene (*gadd45β*, *bax*, *bcl2*, *casp3*), repair related genes (*bmp7*, *flt1*, *vhl* and *fgf23*) and immune-related genes (*pnp*, *ddit4*, *cxcl5*, *vegf-a*, *igfbp3* and *tnf-α*) were significantly upregulated, while the expression levels of other pathway genes were decreased.

### 3.7 Validation of DEGs by qRT-PCR

**FIGURE 6:**
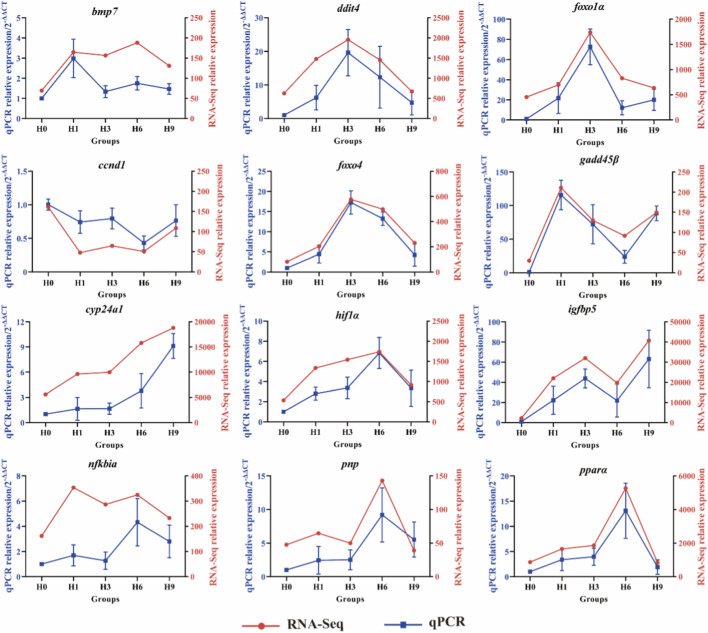
qRT-PCR analysis of 12 genes used to validate RNA-Seq data.

The validation results showed that the expression levels of seven genes (*bmp7*, *foxo4*, *gadd45β*, *cyp24a1*, *hif1α*, *igfbp5*, *nfkbia*) and one gene (*ccnd1*) obtained by qRT-PCR and RNA-Seq were respectively upregulated and downregulated. Although trends in *ddit4*, *foxo1α*, *pnp*, *pparα* expression levels were unclear, qRT-PCR and RNA-Seq revealed consistent changes (Figure 6). The relative expression levels of 12 genes were detected by qRT-PCR and RNA-Seq, and the results were consistent, which supported the validity of the results.

### 3.8 Expression patterns of 12 key DEGs in liver and brain

**FIGURE 7:**
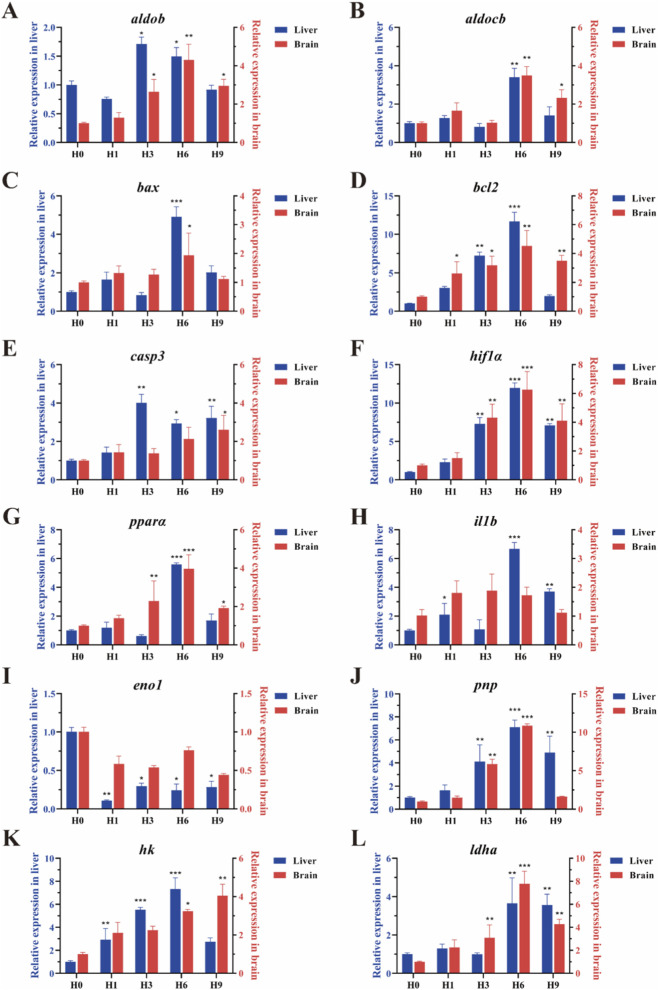
Expression analysis of 12 key DEGs in the liver (blue) and brain (red) of *E. cyanopodus* after hypoxia stress. **(A)** *aldob*; **(B)** *aldocb*; **(C)** *bax*; **(D)** *bcl2*; **(E)** *casp3*; **(F)** *hif1α* **(G)** *pparα* **(H)** *il1b*; **(I)** *eno1*; **(J)** *pnp*; **(K)** *hk*; **(L)** *ldha*. Asterisks indicate significant differences between the control group and hypoxic stress group at each time point. * represents significant differences (*p* < 0.05); ** represents significant differences (*p* < 0.01); *** represents significant differences (*p* < 0.001).

The expression levels of 12 key genes (*aldob*, *aldocb*, *bax*, *bcl2*, *casp3*, *hif1α*, *pnp*, *il1b*, *eno1*, *pparα*, *hk*, *ldh-a*) in the liver and brain were detected and analyzed by qRT-PCR. The results (Figure 7) showed that all genes were significantly expressed under hypoxia for 3 h or 6 h, and some genes were also significantly expressed under hypoxia for 9 h. Apoptosis-related genes such as *bax*, *bcl2* and *casp3* were all upregulated in the liver, reaching their peaks at 6 h, 6 h, and 3 h, respectively. It also showed varying degrees of upregulation in the brain, reaching the peaks at 6 h, 6 h, and 9 h respectively (Figures 7C–E). Immune-related genes *il1b* and *pnp* were significantly expressed in the liver, both reaching their peaks at 6 h *pnp* was significantly expressed in the brain and reached its peak at 6 h, but *il1b* showed no significant differential expression in the brain (Figures 7H,J). In the liver, genes involved in glycolysis/gluconeogenesis such as *aldob*, *pparα*, *hk*, and *ldh-a*, respectively reached their peaks at 3 h, 6 h, 6 h, and 6 h. And in the brain, they reached the peaks at 6 h, 6 h, 9 h, and 6 h, respectively (Figures 7A,G,K,L). *aldocb* was involved in pyruvate metabolism and significantly expressed in both the liver and the brain, with peaks reaching at 6 h in both tissues (Figure 7B). *eno1* showed downregulation under hypoxic stress, with the smallest and most significant expression at 1 h. However, there was no significant differential expression in the brain (Figure 7I). *hif1α* was a key gene in hypoxic stress and significantly expressed in both the liver and brain, reaching their peaks at 6 h (Figure 7F).

### 3.9 Determination of enzyme activities in liver

**FIGURE 8:**
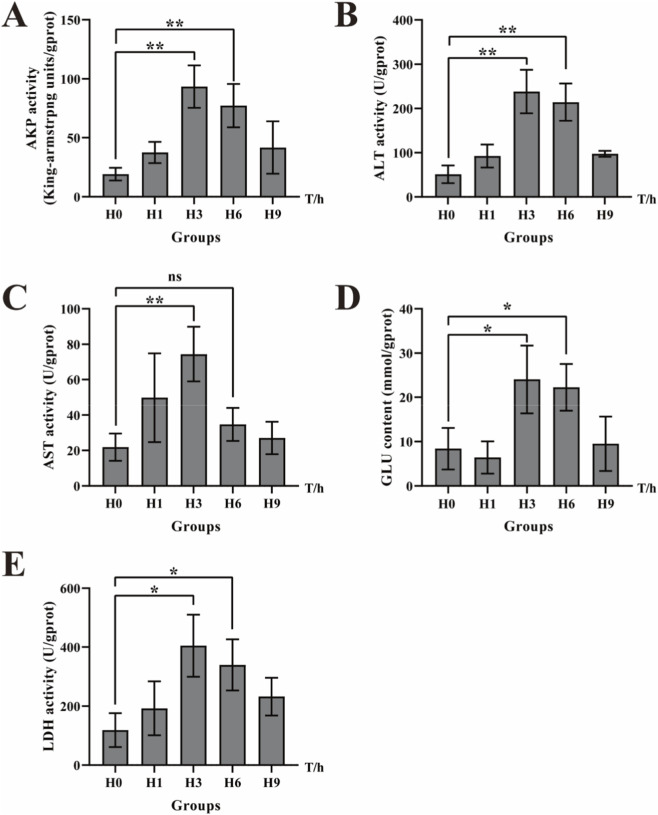
Expressions of liver indexes in *E. cyanopodus* after 9 h hypoxia: AKP **(A)**, ALT **(B)**, AST **(C)**, GLU **(D)** and LDH **(E)**. Asterisks indicate significant differences between the control group and hypoxic stress group at each time point. * represents significant differences (*p* < 0.05), ** represents significant differences (*p* < 0.01).

In order to investigate the effects on glycolysis process and immune-related reaction in *E. cyanopodus* under hypoxic stress, we detected the activities of 5 enzymes. After determining TP in liver tissues, we detected the activities of key metabolic enzymes, including LDH and GLU. The activities of immune-related enzymes such as AKP, AST, and ALT were also detected. As is shown in Figure 8, AKP, ALT and AST all showed a trend of first increasing and then decreasing, the peaks all appeared at 3 h, and the three indexes were respectively 4.87-fold, 4.66-fold and 3.41-fold higher than the H0. At 6 h, they were respectively 4.03-fold, 4.19-fold and 1.59-fold higher than the H0 (Figures 8A–C). GLU and LDH also showed a trend of first increasing and then decreasing, the peaks all appeared at 3 h, and the two indexes were respectively 2.86-fold and 3.41-fold higher than the H0. At 6 h, they were respectively 2.65-fold and 2.86-fold higher than the H0 (Figures 8D,E).

## 4 Discussion

Studies indicate that oxygen depletion in aquatic environments is a common occurrence in fisheries, which can significantly impair various biological functions in fish, including behavioral responses, growth rates, metabolic processes, and immunological competence (Lee et al., 2004; Wang et al., 2023). This study established 1.6 ± 0.05 mg/L dissolved oxygen as the stress experimental condition, we analyzed the expression level of DEGs selected from the liver transcriptome sequencing by qRT-PCR method, the dynamic molecular regulation of hypoxic adaptation was characterized in *E. cyanopodus* across multiple exposure durations. RNA-seq coupled with qRT-PCR validation demonstrated significant enrichment of energy metabolism, immune response and cellular apoptosis pathways along with associated DEGs in the 3 h and 6 h hypoxia-exposed groups.

As a hypoxia-sensitive tissue, the liver plays a crucial role in hypoxic adaptation. Under low-oxygen conditions, liver cells show expression of genes which involve in erythropoiesis, angiogenesis, and glucose metabolism (Baze et al., 2010; Gong et al., 2021). As a vital organ in animals, the brain is extremely sensitive to hypoxia due to it is a high-oxygen-consuming organ. Acute hypoxic stress causes the brain to shift from a normoxic to hypoxic state, causing a series of pathophysiological changes in brain, ultimately leading to irreversible brain damage (Wang M et al., 2021; Welker et al., 2013). Hypoxia-inducible factor (HIF) signaling pathway is an important regulatory pathway for maintaining oxygen homeostasis in tissues and cells under hypoxic stress, affecting oxygen transport and utilization as well as the body’s tolerance to hypoxia, *hif-1α* is a key gene in this pathway (Mandic et al., 2021; Palazon et al., 2014). The hypoxia-inducible gene *hif-1α* activates a complex signaling pathway that promotes liver angiogenesis, thereby ameliorating tissue ischemia, hypoxia, and facilitating liver repair during injury (Kaur et al., 2005). Under acute hypoxic conditions, *hif-1α* expression was markedly upregulated in both *Pelteobagrus fulvidraco* (Wang X et al., 2021) and *Schizothorax prenanti* (Zhao et al., 2020), demonstrating a conserved hypoxic response across these species. The HIF-1 pathway additionally promotes metabolic adaptation through transcriptional activation of anaerobic glycolysis and gluconeogenesis genes, sustaining energy homeostasis during oxygen deprivation (Kim et al., 2006). Several HIF-relative genes, including *aldob*, *eno1*, *ldh-a*, *hk* and *pparα*, participate in the HIF signaling pathway and regulate critical metabolic processes such as glycolysis/gluconeogenesis. There are also some genes involved in pyruvate metabolism, such as *aldocb* and *ldh-a*. Our findings demonstrated significant upregulated of *ldh-a* and *aldocb* in the liver and brain tissues of *E. cyanopodus* following 6 h of acute hypoxia. Notably, *ldh-a* serves as a pivotal enzyme in anaerobic glycolysis, catalyzing the conversion of pyruvate to lactate while generating ATP under hypoxia (Sharma et al., 2022; Valvona et al., 2016). As a key glycolytic enzyme, *aldocb* catalyzes the aldolase reaction, reversibly cleaving fructose-1,6-bisphosphate into glyceraldehyde-3-phosphate and dihydroxyacetone phosphate, a critical step in carbohydrate metabolism (Kawai et al., 2017). In addition, in this study, *aldob* and *pparα* were significantly expressed under acute hypoxic stress, which followed a similar pattern to existing reports (Chen et al., 2019; Shang et al., 2022). These findings indicated that the HIF signaling pathway served as a critical regulatory mechanism governing glucose metabolic adaptation in *E. cyanopodus* under hypoxic conditions.

When fish encounter stressful environments, such as acute hypoxia, they often exhibit a robust inflammatory response. To counteract this, a key repair mechanism involves activating immune defense pathways, which help mitigate tissue damage and enhance survival (Harper and Wolf, 2009; Zheng et al., 2021). It is reported that hypoxic stress significantly elevated hepatocyte apoptosis in both *Carassius gibelio* (Wu et al., 2022a) and *Pelteobagrus vachelli* (Zheng et al., 2021). Through KEGG pathway enrichment analysis (Figures 4B,D) in *E. cyanopodus*, we found that immune-related genes in the H3 and H6 groups were significantly enriched in key signaling pathways, including p53, cAMP, PPAR, PI3K-Akt, and mTOR. The PI3K-Akt signaling pathway stimulates cell proliferation and tumor invasion while upregulating *hif-1α* translation, thereby enhancing *hif-1α*-mediated gene expression. The p53 signaling pathway integrates multiple biological responses, encompassing DNA damage repair, metabolic adaptation, inhibition of angiogenesis, cellular senescence, and programmed cell death (Bieging et al., 2014). *hif-1α* mitigates inflammatory injury by activating the PI3K-Akt, PPAR, and p53 signaling pathways. Additionally, it modulates immune responses, energy metabolism, cell proliferation, and differentiation, thereby promoting tissue repair and enhancing hypoxic adaptation (Abdel-Rahman Mohamed et al., 2019; Spirina et al., 2017). For example, our findings demonstrated that *pnp* and *il1b* participated in immune regulation by maintaining immune cell homeostasis, mitigating inflammatory injury, suppressing apoptosis, and ultimately prolonging survival. Under hypoxic stress, the significant upregulation of *pnp* and *il1b* suggested their potential role in mitigating tissue inflammatory damage induced by hypoxia. Furthermore, *hif-1α* activates the apoptosis-regulating genes *bax* and *bcl2*, which function downstream of the p53 signaling pathway to coordinately control apoptotic processes (Wang et al., 2019). Under conditions of acute hypoxia, organisms upregulate *bcl2* expression as an adaptive mechanism to inhibit apoptotic pathways (Shroff et al., 2007). *bax* as a key member of the *bcl2* gene family, encodes one of the most critical pro-apoptotic proteins. The BAX protein functions by forming heterodimers with BCL-2, thereby serving as its primary antagonist in apoptotic regulation (Jin et al., 2011). Studies indicate that the BCL-2/BAX protein ratio serves as a critical determinant of apoptotic regulation. A decreased BCL-2/BAX ratio results in *bax* dominance, facilitating the efficient clearance of damaged cells in fish (Gu et al., 2023). The dynamic balance between BCL-2 and BAX, reflected in their expression ratio, mediates their functional antagonism. This regulatory mechanism promotes appropriate apoptotic responses to environmental stress while preventing excessive cell death, thereby maintaining cellular homeostasis. These findings align with the gene expression patterns observed in our study, demonstrating that coordinated immune responses and apoptotic regulation work synergistically to maintain organismal homeostasis under hypoxic stress.

Fish undergo a series of intricate biochemical reactions to adapt to the physiological changes induced by hypoxic stress (Abdel-Tawwab et al., 2019). Hypoxia promotes oxidative stress, impairs antioxidant defenses, and inflicts substantial harm to cell (Lushchak, 2011; Nitz et al., 2020; Yang et al., 2017). Since ALT and AST exhibit a positive correlation with hepatocyte lysis, increased enzymatic activity suggests mild cellular damage or stress-induced tissue injury. Consequently, these enzymes are well-established biomarkers for assessing liver function impairment (Akbary et al., 2018; Dassarma et al., 2018; Palanivelu et al., 2005). Because of the liver injury, the disruption of hepatocyte integrity leads to a marked increase in ALT and AST activity (Sheikhzadeh et al., 2012). In this study, hypoxic stress induced a significant increase in hepatic AST and ALT activity in *E. cyanopodus*, which has also been reported in *Hyphessobrycon callistus* (Pan et al., 2009). AKP contributes to physiological protection and immune defense, while engaging in multiple metabolic pathways such as detoxification, metabolism, and biosynthesis of macromolecules (Chen et al., 2022; Suzuki and Mori, 1990). The activity profile of AKP shows significant variation depending on stressor type, with distinct modulation patterns observed under thermal, acidic, hypoxic, and heavy metal stress conditions (Chen et al., 2007). Therefore, AKP is often used as an indicator to evaluate the immune status of an organism (Yang S et al., 2019). Increased AKP activity serves as a biochemical marker of compromised membrane permeability and structural integrity, ultimately resulting in cellular damage and subsequent hepatobiliary inflammation in the host organism (Lin et al., 2015). In the present study, hypoxic stress induced a significant elevation of hepatic AKP activity in *E. cyanopodus*, indicative of severe inflammatory liver injury. These findings demonstrated that hypoxic conditions promote hepatic oxidative damage and trigger immune activation (Jia et al., 2023).

Blood glucose concentration is highly sensitive to environmental fluctuations, and hypoxic stress fundamentally shifts the energy metabolism paradigm in fish from oxidative phosphorylation toward enhanced glycolysis/gluconeogenesis and pyruvate metabolic pathways (Ding et al., 2020; Qi et al., 2018; Wu et al., 2022b; Yang C et al., 2019). During anaerobic glycolysis, carbohydrates are initially hydrolyzed to glucose, which is subsequently converted by lactate dehydrogenase (LDH) into pyruvate as the primary metabolic intermediate. Pyruvate is then reduced to lactate while generating ATP (Mizock, 1995), with the accumulated lactic acid serving as a crucial component of energy metabolism through anaerobic respiration under environmental stress conditions (Wang M et al., 2021; Xiaolong et al., 2018). The elevated LDH activity demonstrates its pivotal role in anaerobic metabolism, while also facilitating the gluconeogenic conversion of lactate to pyruvate for glucose synthesis (Mahfouz et al., 2015). These findings revealed that the respiratory and metabolic patterns of *E. cyanopodus* were changed under hypoxic stress, primarily through upregulation of glycolytic, gluconeogenic, and pyruvate metabolic pathways as an adaptive strategy.

## 5 Conclusion

In this study, we employed RNA-seq, qRT-PCR, and physiological and biochemical analyses to investigate the response of *E. cyanopodus* to hypoxic stress. Transcriptomic profiling of liver tissues at different time points (H0, H1, H3, H6, and H9) revealed a total of 6152 DEGs across the five experimental groups. qRT-PCR validation confirmed that several key DEGs were significantly expressed under hypoxic stress, with 3–6 h of hypoxia emerging as the critical period in the stress response. KEGG pathway enrichment analysis revealed that DEGs in the H3 and H6 groups were predominantly enriched in energy metabolism and immune response pathways. Key energy metabolism pathways included glycolysis/gluconeogenesis, pyruvate metabolism, glutathione metabolism, and pyrimidine metabolism. Meanwhile, immune-related pathways such as p53 signaling, PPAR signaling, and PI3K-Akt signaling pathways were primarily associated with innate immunity and the regulation of apoptosis. We selected the significantly expressed DEGs from these pathways and examined their expression patterns in both liver and brain tissues. It could be concluded that these DEGs were significantly expressed in both liver and brain, and most of these DEGs had the highest expression levels at 3 h or 6 h. Furthermore, hypoxic stress altered the activities of glycolytic and immune-related enzymes in the liver, subsequently influencing the expression of genes associated with energy metabolism and inflammatory responses. These findings exactly confirm that 3–6 h of hypoxia is the key duration for *E. cyanopodus*. It also suggests that *E. cyanopodus* adapts to hypoxic conditions by enhancing anaerobic glycolysis as a key energy-producing pathway, while simultaneously activating immune defenses and apoptotic mechanisms to maintain metabolic and cellular homeostasis. In conclusion, this study elucidates key molecular mechanisms underlying hypoxia tolerance in *E. cyanopodus*, providing novel insights that advance our understanding of marine fishes’ physiological responses to acute hypoxic stress.

## Data Availability

The RNA-Seq raw reads data presented in the study are deposited in the NCBI repository, accession number PRJNA1320850.
